# Superconductivity mediated by polar modes in ferroelectric metals

**DOI:** 10.1038/s41467-020-18438-0

**Published:** 2020-09-25

**Authors:** C. Enderlein, J. Ferreira de Oliveira, D. A. Tompsett, E. Baggio Saitovitch, S. S. Saxena, G. G. Lonzarich, S. E. Rowley

**Affiliations:** 1 Cavendish Laboratory, University of Cambridge, J. J. Thomson Avenue, Cambridge, CB3 0HE UK; 2 Centro Brasileiro de Pesquisas Físicas, Rua Dr Xavier Sigaud 150, Rio de Janeiro, 22290-180 Brazil; 3 UFRJ, Estrada de Xerém 27, Xerém, Duque de Caxias, Rio de Janeiro 25245-390 Brazil; 4 National University of Science and Technology MISiS, Leninsky Prospekt 4, Moscow, 119049 Russia

**Keywords:** Electronic devices, Ferroelectrics and multiferroics, Phase transitions and critical phenomena, Superconducting properties and materials

## Abstract

The occurrence of superconductivity in doped SrTiO_3_ at low carrier densities points to the presence of an unusually strong pairing interaction that has eluded understanding for several decades. We report experimental results showing the pressure dependence of the superconducting transition temperature, *T*_*c*_, near to optimal doping that sheds light on the nature of this interaction. We find that *T*_*c*_ increases dramatically when the energy gap of the ferroelectric critical modes is suppressed, *i.e*., as the ferroelectric quantum critical point is approached in a way reminiscent to behaviour observed in magnetic counterparts. However, in contrast to the latter, the coupling of the carriers to the critical modes in ferroelectrics is predicted to be small. We present a quantitative model involving the dynamical screening of the Coulomb interaction and show that an enhancement of *T*_*c*_ near to a ferroelectric quantum critical point can arise due to the virtual exchange of longitudinal hybrid-polar-modes, even in the absence of a strong coupling to the transverse critical modes.

## Abstract (web summary)

Superconductivity in doped SrTiO_3_ near to a ferroelectric quantum critical point emerges due to a strong interaction driving the formation of Cooper pairs, the nature of which has remained elusive for several decades.  Here, the authors reveal that pairing is due to the exchange of longitudinal hybrid polar modes rather than transverse critical modes.

## Introduction

**Fig. 1:**
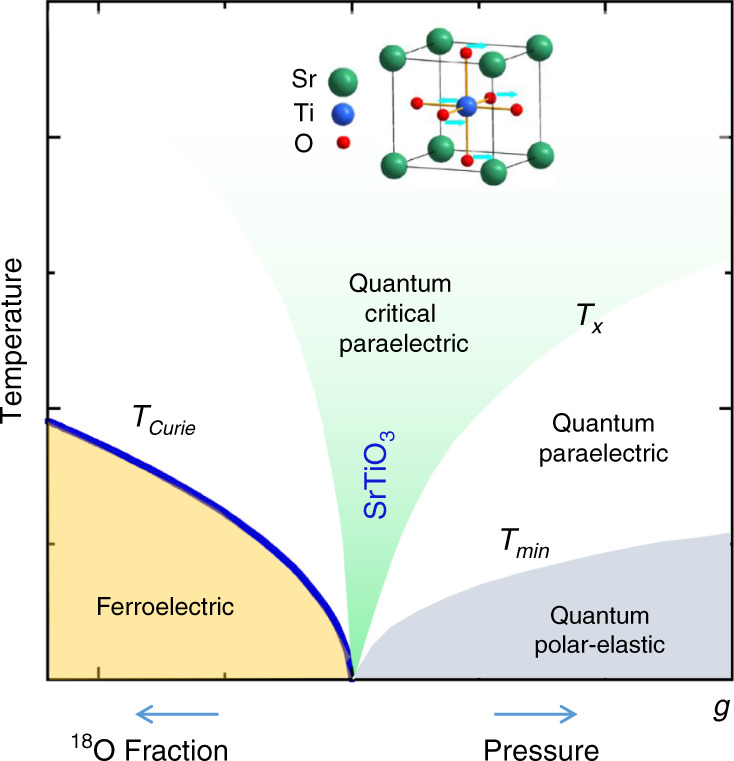
The phase diagram of SrTiO_3_ based on a quantum *ϕ*^4^ self-consistent field model of a displacive ferroelectric^3^. The vertical axis is the temperature while the horizontal axis represents a quantum tuning parameter, *g*, that can be varied, for example, by the application of hydrostatic pressure, isotopic substitution as in SrTi(^16^O_1−*y*_^18^O_*y*_)_3_, or by chemical substitution as in the cases of Sr_1−*y*_Ca_*y*_TiO_3_ and Sr_1−*y*_Ba_*y*_TiO_3_. In strontium titanate *T*_Curie_ and *T*_min_ are, respectively, of order 25 K at 0 kbar and *x* = 1, and 8 K at 10 kbar and *x* = 0. The green shaded region highlights the quantum critical regime, characterized by an approximately quadratic temperature dependence of the inverse dielectric function as seen experimentally in SrTiO_3_, ^18^O substituted SrTiO_3_, KTaO_3_, and other materials. The curve *T*_min_ marks the temperature of the minimum in the inverse dielectric susceptibility as observed and predicted by a model involving the electrostrictive coupling of polarization and strain and below which the system forms a quantum polar-elastic regime^3,S7 4^. An extension of the phase diagram would include an additional orthogonal axis representing the charge carrier density, *n*, of doped or injected electrons which gradually supresses ferroelectricity. The introduction of carriers results in the emergence of a dome of superconductivity mediated by longitudinal hybrid polar modes near to the quantum critical point under specific conditions as observed in the model and experiments presented in the main text.

Strontium titanate is an incipient ferroelectric insulator, which can be tuned essentially continuously into the ferroelectric phase via a ‘quantum’ tuning parameter, such as chemical substitution, isotopic substitution or applied stress (see, e.g., refs. ^1–7^). The temperature-quantum tuning parameter phase diagram of SrTiO_3_ and related materials has recently been discussed in terms of a phenomenological model involving the interaction of the local ferroelectric order-parameter field with itself, and with the strain field of the lattice^3,S1–S13^ (see Fig. 1). A self-consistent perturbative treatment of the model in an isotropic approximation leads to an accurate quantitative description of the phase diagram and the temperature dependence of the uniform dielectric function, *ε*_0_, without the use of freely adjustable parameters^3^. The phase diagram is characterized by (i) a ferroelectric transition temperature *T*_Curie_ that terminates at a quantum critical point (QCP), (ii) a low-temperature crossover curve *T*_*x*_ separating a power law (1/*ε*_0_ ~ *T*^2^) and an exponential temperature dependence of 1/*ε*_0_ also terminating at the QCP, and (iii) a high-temperature crossover curve separating classical (Curie–Weiss) and quantum behaviour of the temperature dependence of 1/*ε*_0_. An additional crossover curve *T*_min_ that terminates at the QCP marks the position of a minimum in the temperature dependence of 1/*ε*_0_, which arises from the coupling of the electrical polarization to the lattice strain field^3,S7,S14–S17^. More exotic behaviour beyond that indicated above^S8,S11,S18^ is anticipated at least for sufficiently low frequencies and low temperatures below that probed experimentally thus far. Quantum phase transitions have also been observed in a diversity of different ferroelectrics including in oxides^S19^, organics^S14^, hydrogen-bonded crystals^S20^, electronic ferroelectrics^S21^ and multiferroics^S15^ and have been highlighted in recent reviews^S22^ ^8^.

The substitution of Ti by Nb leads to an extra electron per substituted unit cell of SrTiO_3_, which is bound to Nb only very weakly due to the high dielectric constant of the host lattice. For a doped electron density, *n*, above the order of 10^15^ cm^−3^ the doped electrons are promoted to the *t*_2g_ bands that are split by the spin–orbit interaction as well as by a structural perturbation observed below ~100 K of the starting simple cubic structure of SrTiO_3_^S23^. Charge carriers may also be introduced by lanthanum doping, oxygen reduction, interface engineering and electrostatic gating. The introduction of charge carriers leads to an additional axis (*n*) in the ferroelectric phase diagram of Fig. 1, which is available for exploration. The carriers in SrTiO_3_ (conduction electrons) occupying the lowest of these *t*_2g_ bands tend to dominate the properties of principal interest in the discussion below, but we note that additional effects due to the progressive filling of the three *t*_2g_ bands have been reported^S24^. Umklapp processes may be neglected under our conditions as well as inter-valley scattering processes that would require the presence of multiple Fermi surface pockets well separated in the Brillouin zone. These are not present in electron-doped SrTiO_3_ as confirmed by several quantum oscillation experiments and band structure calculations (see, for example, refs. S23 and S25 and Supplementary Information). Cooper-pair formation and superconductivity at such low carrier-concentrations are considered by many investigators to be remarkable even after half a century since its observation in SrTiO_3_, the first of the oxide superconductors^9^. Despite numerous investigations, the detailed nature of the relevant effective interaction mediating superconductivity in carrier-doped SrTiO_3_^S24,S26-S31^ ^9–25^ and related systems such as KTaO_3_^26^, LaAlO_3_/SrTiO_3_ interfaces^19,27^, ferroelectric Ca_*x*_Sr_1−*x*_TiO_3−*δ*_^28^ and FeSe/SrTiO_3_^29^, continues to be debated.

We have investigated specimens with carrier densities, *n*, in the range 2.0 × 10^18^–4.0 × 10^20^ cm^−3^ spanning the superconducting dome maximum of the temperature-carrier density phase diagram of SrTiO_3_ with Nb substitution (see “Methods” section and Supplementary Information Fig. S1). In this work, we present results showing the pressure dependence of the superconducting transition temperature and develop a superconductivity model appropriate for electron-doped SrTiO_3_ and related materials.

## Results

### Electron-doped SrTiO_3_ under pressure

**Fig. 2:**
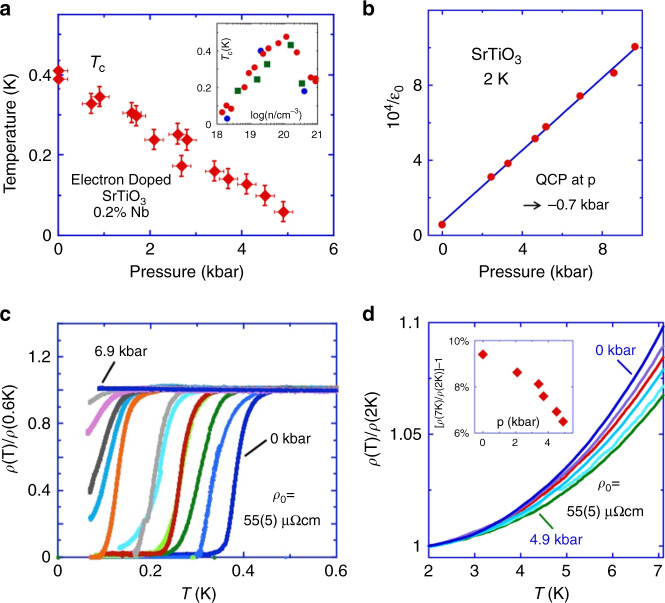
The observed superconductivity temperature–pressure phase diagram for Nb-doped SrTiO_3_. **a** Superconducting transition temperature, *T*_c_, as determined from resistivity data vs. pressure for a SrTi_1-*x*_Nb_*x*_O_3_ sample (see “Methods” section) with a carrier density depending on *x* near the dome maximum of *T*_c_ vs. *n* shown in the inset (red circles are from S27, green squares are from S23 and blue circles are our data, Fig. S1). **b** Pressure dependence at low *T* of the inverse dielectric constant 1/*ε*_0_, proportional to Ω(0)^2^, for the undoped state^4,6,S16,S17^. Ω(0) is only slightly changed (see Eq. (10)) up to carrier concentrations of around 10^19^ cm^−3 S34^. **c** *T* dependence of the resistivity, *ρ*, below 1 K at different pressures in the same sample as in **a**. *ρ* is scaled to the normal state residual value, *ρ*_0_, which is weakly dependent on pressure. *T*_c_ in **a** is defined from the 10% drop of *ρ* from *ρ*_0_. For decreasing values of *T*_c_, *p* = 0, 0 (after decompression), 0.9, 1.6 1.7, 2.6, 2.8, 3.4, 3.7, 4.1, 4.5, 4.9, 6.9 kbar, respectively. **d** *T* dependence of *ρ* above 1 K at different pressures scaled to *ρ*_0_. The pressures may be inferred from the inset, which shows the relative change of the *T*^2^ resistivity coefficient (see caption of Fig. S1) vs. pressure. The collapse of *T*_c_ with increasing pressure and hence 1/*ε*_0_ is seen to be extraordinarily rapid, pointing to a significant growth of the pairing strength on approaching the ferroelectric quantum critical point.

In Fig. 2a, c we present the pressure dependence of the superconducting transition temperature, *T*_c_, as determined from resistivity measurements in a sample with nominal 0.2 at.% Nb doping (see “Methods” section), which has an intermediate carrier density near that of the dome maximum where *T*_c_ is as high as 0.4 K. As shown in these figures we find that *T*_c_ drops sharply with modest pressures and collapses towards absolute zero above 5 kbar. Thus, *T*_c_ increases with decreasing 1/*ε*_0_ (Fig. 2b) or decreasing gap of the soft transverse-optical polar phonon frequency^6,S1-S3,S32,S33^, Ω(*q*), connected with the ferroelectric QCP. Ω(*q*) is relatively weakly dependent on *n* up to of the order of 10^19^ cm^−3^ ^S34,S35^ ^30^ and its *n* dependence is accounted for in the model calculations that follow. This behaviour is reminiscent of the increase of *T*_c_ observed on approaching magnetic QCPs in nearly magnetic metals^31^.

This finding is consistent with other strain and pressure measurements tuning Ω(*q*) over more limited ranges^32–34^, as well as recent reports of increases of *T*_c_ observed upon calcium^28^ and oxygen-18 isotope^35^ substitutions. It is also supported by the observation of a lower value of *T*_c_ in electron-doped KTaO_3_^26^, which is a quantum paraelectric further away from the QCP (having 1.5 times the value of Ω(*q* = 0) at low temperature compared to SrTiO_3_ in the undoped starting material^3^). Note that the ‘ferroelectric’ transition in the conducting state^36^, where the uniform static dielectric function is strictly singular for all finite *n*, is defined by the condition Ω(*q*) → 0 at small *q*, combined with an inversion symmetry-breaking local polar distortion. The symbol *ε*_0_ is defined here as the starting uniform static dielectric function for the undoped state.

We also comment that in the normal state the resistivity varies approximately as the square of the temperature with a *T*^2^ coefficient that changes by ~30% in the pressure range where *T*_c_ drops by an order of magnitude or more (Fig. 2a, d). This finding provides a new constraint on the theory of the *T*^2^ variation of the resistivity in SrTiO_3_^37,38^.

### Superconductivity mediated by longitudinal hybrid-polar-modes

The above findings and our pressure measurements shown in Fig. 2a also provide further constraints on models for the mechanisms for carrier pairing in these systems. In light of the apparent enhancement of *T*_c_ on approaching a ferroelectric QCP, it is natural to consider first the role of the polar optical modes consisting of a multiplet of transverse and longitudinal components, the lowest transverse mode frequency Ω(*q*) as defined above, vanishing at the ferroelectric transition at *q* = 0. Analyses suggest that for the model described here it is sufficient to consider the highest longitudinal optical (LO) polar mode, Ω_LO_, which is ~100 meV in the starting undoped state and weakly *q* dependent, and the lowest transverse optical (TO) polar mode, Ω(*q*), which can be one or more orders of magnitude lower than Ω_LO_, varying as $1/\sqrt {\varepsilon _0}$, in the insulating state. As shown in detail for example by Ruhman and Lee in the appendix to ref. ^21^, the contribution to the effective paring interaction of the intermediate TO and LO modes tend to cancel out in first approximation.

In terms of the dielectric screening model^S36 ^^16,39–42^, the above effective pairing interaction in the low *n* limit is attractive in the frequency range Ω(*q*) < *ω* < Ω_LO_, which extends to *ω* = 0 at low *q* when *ε*_0_ diverges (Ω(*q*) → 0) at the ferroelectric critical point (model outlined below). In this limit the induced interaction arises from the coupling of the conduction electrons with fluctuations of the lattice of ions leading to an effective coupling strength that can be significantly larger than that of a lattice of essentially neutral atoms as in conventional phonon-mediated superconductivity. At higher *n* the doped conduction electrons can screen the dipolar interactions leading to a collapse of the attractive interaction and hence of *T*_c_, in agreement with observation (inset of Fig. 2a).

It has been suggested that the mediation of polar optical modes, while of key importance in reducing the Coulomb repulsion as *ε*_0_ increases, might be supplemented by an additional pairing mechanism to account for superconductivity or at least for a quantitative understanding of the magnitude of *T*_c_. A number of additional candidates have been proposed involving: (i) residual coupling to critical TO modes missing in the dynamical screening model^20,43,44^; (ii) plasmons in the conduction electron system^21^, which must be included for minimal consistency of any proposed model; (iii) multi-valley transition processes^9,13^; (iv) non-polar acoustic phonons (see, e.g., the Appendix in ref. ^21^; (v) non-polar soft optical phonons^12^; (vi) currents associated with transverse polar optical modes; (vii) non-cancelling contributions of polar optical modes in between Ω(*q*) and Ω_LO_^21^; (viii) phonon modes localized around the dopant impurity sites^23^; (ix) effects associated with Sr disordering at low temperatures^45^; and other distinct models, e.g., refs. ^46–48^. Theories involving the formation of polarons and bipolarons^14^, as well as pre-formed pairs^10^, have also been considered. Though of interest in their own right these mechanisms have not been shown by quantitative analyses free of adjustable parameters to play central roles in understanding superconductivity in the case of SrTiO_3_.

Having investigated all of these proposed theories in light of realistic model parameters for SrTiO_3_, and our new experiments, we consider here a minimal description that includes pairing due to the dipolar fluctuations of the coupled ion (polar optical modes) and charge-carrier system in a dielectric screening model of the effective long-wavelength interaction between carriers expressed in the form

1 $$V\left( {q,\omega } \right) = \frac{{4\pi e^2}}{{q^2\varepsilon (q,\omega )}} = \frac{{4\pi e^2}}{{q^2}}\left( {1 - \frac{{4\pi \chi (q,\omega )}}{{1 + 4\pi \chi (q,\omega )}}} \right)$$

where $\chi \left( {q,\omega } \right) = \varepsilon \left( {q,\omega } \right) - 1$ is the wavevector and frequency-dependent dielectric susceptibility and *q* and *ω* measure, respectively, the momentum and energy transfers in two-electron scattering processes. In these calculations we adopt units in which *ħ* = *k*_B_ = 1. The approach as set out below using the interaction given by Eq. (1) builds on the foundational work of Gurevich et al.^39^ and Takada^16,49^.

By way of contrast the pairing interaction due to the virtual exchange of magnetic fluctuations on the border of a ferromagnetic QCP in the simplest case for parallel spins is given by *V*_m_(*q*, *ω*) = −*g*^2^*χ*_m_(*q*, *ω*), where *g* is a coupling parameter and *χ*_m_(*q*,*ω*) is the wavevector and frequency-dependent *magnetic* susceptibility (see, e.g., ref. ^50^). The model defined by Eq. (1) differs in including a screening of the induced response (second term) and a direct repulsion (first term), which tend to cancel at the TO mode frequencies and in particular for the critical soft mode frequency associated with a ferroelectric QCP. In this case the attractive interaction arises in terms of hybrid LO modes as discussed below.

We assume that for each mode of wavevector *q*, *χ*(*q*, *ω*) can be described by simple oscillator contributions from fluctuations of the dipolar density of the ions and the contribution for the carriers given by the Lindhard function (see, e.g., refs. ^16,42,50^) as given approximately for low *q* by

2a $$\frac{{\varepsilon (q,\omega )}}{{\varepsilon _\infty }} = 1 + \frac{{{\mathrm{{\Omega} }}_{\rm{{p}}}^2}}{{{\mathrm{{\Omega} }}\left( q \right)^2 - \omega ^2}} + \chi _{{\rm{{el}}}}(q,\omega )$$

where Ω_p_ is the bare plasma frequency for the ions in a medium with background dielectric constant *ε*_∞_,

2b $${\mathrm{{\Omega} }}\left( q \right) = \sqrt {{\mathrm{{\Omega} }}\left( 0 \right)^2 \, +\, v_{\rm{{s}}}^2q^2}$$

is the bare frequency spectrum at low *q* for ionic fluctuations characterized by a frequency gap, Ω(0) and a velocity, *v*_s_, and *χ*_el_(*q*, *ω*) reduces to −(*ω*_p_/*ω*)^2^ and (ω_*p*_/ω(*q*))^2^, respectively, for *ω*(*q*) >> *ω* and *ω*(*q*) << *ω,* where *ω*_p_ is the bare plasma frequency for the carriers and

2c $$\omega \left( q \right) = v_{\rm{F}}q/\sqrt 3$$

is the bare characteristic frequency at low *q* for density fluctuations in the carrier system with Fermi velocity *v*_F_. *χ*_el_(*q*, *ω*) can be expressed approximately by an interpolation formula^21,23^ similar in form to the second term on the right-hand side of Eq. (2), but in principle including effects of dissipation and a restriction to *q* below of the order of the Fermi wavevector, *k*_F_, as defined by the Lindhard function. (The role of dissipation missing in the simplest interpolation model for *χ*_el_(*q*, *ω*) is discussed under “Methods” section.) Ω(*q*) and *ω*(*q*) are the two transverse mode frequencies where *ε*(*q*, *ω*) becomes large and the interaction *V*(*q*, *ω*) weak.

For the simplest interpolation model for *χ*_el_(*q*, *ω*) the inverse of *ε*(*q*, *ω*) can also be expressed in terms of the sum of two resonance modes so that the effective interaction *V*(*q*, *ω*) defined in Eq. (1) can be written using Eqs. (2a–2c) in the form

3 $$V\left( {q,\omega } \right) = \frac{{4\pi e^2}}{{q^2\varepsilon _\infty }}\left( {1 - \frac{{\gamma _ - \omega _ - \left( q \right)^2}}{{\omega _ - \left( q \right)^2 {\,}-{\,} \omega ^2}} - \frac{{\gamma _ + \omega _ + (q)^2}}{{\omega _ + (q)^2 - \omega ^2}}} \right)$$

Here *ω*_±_(*q*) are the coupled carrier-ion longitudinal frequencies (hybrid longitudinal frequencies), defined by the condition *ε*(*q*, *ω*) → 0, and *γ* ± (*q*) are the corresponding coupling parameters, all determined straightforwardly by the starting material parameters defining *ε*(*q*, *ω*) in Eq. (2). In contrast to the starting transverse modes, the longitudinal hybrid mode frequencies, *ω* ± (*q*), correspond to cooperative motion of the charge carriers and the ions. We postulate that these hybrid longitudinal modes are the dominant source of attraction between conduction electrons.

**Fig. 3:**
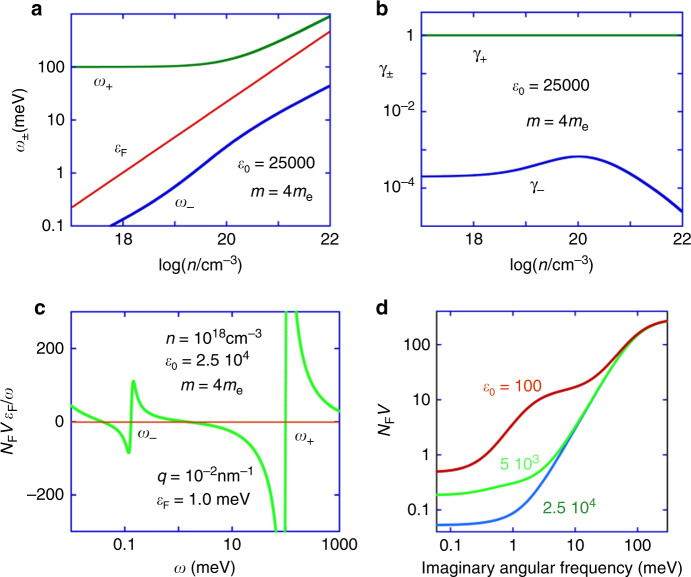
The calculated longitudinal hybrid polar optical frequencies and effective interaction between doped carriers for SrTi_1-*x*_Nb_*x*_O_3_ (Nb-doped SrTiO_3_). **a**, **b** The longitudinal hybrid frequencies *ω*_±_(*q*), Fermi energy, *ε*_F_, and coupling functions *γ*_±_(*q*) vs. carrier density, *n*, defined by Eqs. (1)–(3) and materials parameters relevant to SrTiO_3_ (see “Methods” section) ℏ = *k*_B_ = 1). **c** The normalized effective interaction *N*_F_*V*(*q*, *ω*)*ε*_F_/*ω* vs. *ω* for a representative carrier density, *n*, and a low value of *q* << *k*_F_, where *N*_F_ is the density of states of the doped carriers at the Fermi level and *V*(*q*, *ω*) is defined by Eqs. (1)–(3). The TO frequency and effective mass for the lower *t*_2g_ band, *m*, are set to representative values (see “Methods” section). The *n* dependences of these quantities become significant at high *n* and are included in the calculations of *T*_c_ presented in Fig. 4 (see “Methods” section). **d** The interaction as in **c** plotted against imaginary frequency within the Matsubara formalism used in the numerical calculations. Similar curves for two lower values of *ε*_0_ are also shown. The two steps most clearly visible for the case *ε*_0_ = 100 are associated with the two hybrid LO modes. An increase of *ε*_0_ increases the size of the upper step, but decreases the size of the lower step pointing essentially to an inversion of the trend of pairing strengths vs. *ε*_0_ as illustrated in Fig. S4.

At a low carrier density, *n*, where *ω*_p_ << Ω_p_ the lower hybrid longitudinal mode of frequency *ω*_−_(*q*) < *ω*_p_ can be thought of as a carrier plasma mode as screened by the ions, so that *ω*_−_(*q*) can be much lower than *ω*_p_ (and even *v*_F_*k*_F_), while the upper longitudinal hybrid mode of frequency *ω*_+_(*q*) can be described as a weakly renormalized LO polar phonon mode. On the other hand at high carrier density, *n*, where *ω*_p_ >> Ω_p_ the lower longitudinal hybrid mode of frequency *ω*_−_(*q*) < *ω*_p_ can be thought of as a polar optical phonon mode as screened by the charge carriers, so that *ω*_−_(*q*) tends to Ω(*q*), while the upper longitudinal hybrid mode of frequency *ω*_+_(*q*) can be described as a weakly screened carrier plasma mode. The *n* dependences of *ω*_±_(*q*) are illustrated in Fig. 3a for starting parameters relevant to SrTiO_3_ (see “Methods” section).

Equally significant are the coupling functions *γ*_−_(*q*) and *γ*_+_(*q*) (Fig. 3b), which affect the resonance weights in Fig. 3c, d that show the calculated real part of the normalized retarded interaction potential based on Eqs. (1)–(3) versus real and imaginary frequencies, respectively. The coupling parameter *γ*_−_(*q*), corresponding to the lower longitudinal hybrid mode, tends to be suppressed by screening with increasing *ε*_0_. At low *n* (*ω*_p_ << Ω_p_), where the upper longitudinal hybrid frequency tends to be far above *v*_F_*k*_F_ (Fig. 3a) and hence ineffective in pairing, the suppression of *ω*_−_(*q*) along with *γ*_−_(*q*) tends to reduce *T*_c_ with increasing *ε*_0_. At high *n* (*ω*_p_ >> Ω_p_), where the upper longitudinal hybrid frequency *ω*_+_(*q*) is not far above *v*_F_*k*_F_ (Fig. 3a) and hence potentially important in pairing, the suppression of *γ*_−_(*q*) and *ω*_−_(*q*) tends to open up a wide region of attraction up to *ω*_+_(*q*) that enhances rather than suppress superconductivity with increasing *ε*_0_, i.e., on approaching the ferroelectric QCP. Note that the usual Bardeen–Pines form of the interaction often used in calculations of conventional phonon-mediated superconductivity is a special limit of Eq. (3) as explained later and shown in Eq. (7b).

The conditions favourable for superconductivity can also be observed by looking at the plot of the interaction versus imaginary frequency within the Matsubara formalism as shown in Fig. 3d. The curves comprise three plateaus each separated by a step in *N*_F_*V*, one at higher frequencies and the other at lower frequencies. The *N*_F_*V* step size at higher frequencies increases as *ε*_0_ increases which leads to an enhancement of superconductivity. On the other hand, the step size at lower frequencies decreases as *ε*_0_ increases reducing the propensity of the system to forming a superconducting state. The relative importance of these two effects depends on the Fermi energy and correspondingly the carrier density. If the Fermi energy (shown in Fig. 3a) is near to that of the position of the upper step, then the upper step plays a dominant role, i.e., superconductivity is enhanced when the system is tuned closer to the QCP. If the Fermi energy is near to that of the position of the lower step, then the effects of the lower step become dominant, i.e., superconductivity can be suppressed when tuning the system closer to the QCP.

In an attempt to gain more detailed insight on the conditions favourable for superconductivity for the pairing interaction defined by Eqs. (1)–(3) we examine the predictions of the spin-singlet BCS^S38-S41^ gap equation defining *T*_c_ in the weak coupling approximation^S39^, which is appropriate for the parameters relevant to SrTiO_3_,

4 $${\mathrm{{\Delta} }}\left( k \right) = \mathop {\sum}\limits_{k{^\prime}} {U(k,k{^\prime})\frac{{{\mathrm{{\Delta} }}k{^\prime}}}{{2\xi (k{^\prime})}}{\rm{{tanh}}}\left( {\frac{{\xi (k{^\prime})}}{{2T_{\rm{{c}}}}}} \right)}$$

where Δ(*k*) is a superconductivity gap function, *ξ*(*k*) is the one-electron energy spectrum measured from the Fermi level, and *U*(*k, k*′) is an appropriate interaction kernel to be defined below and in “Methods” section. We assume that the band can be treated in an effective mass approximation with an effective mass, *m*, which in general depends o*n n*.

It is sometimes assumed that the kernel can be approximated at least in the weak coupling limit by the interaction *V*(*q*, *ω*) with *q* = *k*′ − *k* and *ω* = *ξ*(*k*′) − *ξ*(*k*) equal to the momentum and energy transfers, respectively, in a process in which a pair of electrons in one-particle states with wavevectors *k* and −*k* scatter into states with wavevectors *k*′ and −*k*′. However, this ‘on-shell’ approximation does not fully take into account the way in which the dynamics of the interacting charge carriers affect the total relevant interaction. For example, in the on-shell approximation the effective pairing interaction is independent of the velocities of the interacting carriers when the two velocities are equal, which is inconsistent with the intuitive expectation that the polarization of the medium produced by moving carriers should be dependent on their velocities. Also excluded are the effects of changes in vacuum fluctuations due to changes in the occupation of the one-particle states that accompany each pair transition (−*k*, *k*) → (−*k*′, *k*′). To avoid such limitations we begin by using the Eliashberg equations^S41^, to determine the kernel *U*(*k*, *k*′) in Eq. (4). The key equation is that shown below (Eq. (5)) with *V* as defined in Eq. (3).

5 $${\mathrm{{\Lambda} }}\left( T \right){\mathrm{{\Phi} }}\left( {{\boldsymbol{p}},i\omega _n} \right) = \frac{T}{N}\mathop {\sum }\limits_{{\mathrm{{\Omega} }}_n,{\boldsymbol{k}}} V({\boldsymbol{p}} - {\boldsymbol{k}},i\omega _n - i{\mathrm{{\Omega} }}_n)\left| {G({\boldsymbol{k}},i{\mathrm{{\Omega} }}_n)} \right|^2{\mathrm{{\Phi} }}\left( {{\boldsymbol{k}},i{\mathrm{{\Omega} }}_n} \right)$$

Here $G({\boldsymbol{k}},i{\mathrm{{\Omega} }}_n)$ is the one-particle Green’s function for the conduction electrons, ${\mathrm{{\Phi} }}\left( {{\boldsymbol{p}},i\omega _n} \right)$ the anomalous self-energy, *N* is the number of allowed wavevectors in the Brillouin zone, and ${\mathrm{{\Lambda} }}\left( T \right) = 1$ as *T* → *T*_c_. These and the remaining terms are defined more fully in the discussion of Eq. (A6) in ref. ^50^. In the weak-coupling limit for the parameters relevant to SrTiO_3_ the frequency summation in Eq. (5) can be carried out analytically. This leads to a kernel shown in “Methods” section and investigated in detail by Kirzhnits, Maksimov and Khomskii (KMK)^51^, originally applied to the case of SrTiO_3_ by Takada^16^. The KMK kernel *U*(*k*, *k*′) differs significantly from *V*(*q*, *ω*), but is fully specified by it. More precisely *U*(*k*, *k*′) is given by a particular average along the imaginary frequency axis of the continuation of *V*(*q*, *ω*) in the complex frequency plane (see “Methods” section). Importantly, *U*(*k*, *k*′) depends on the absolute values of the velocities of the interacting carriers and not on the velocity difference assumed in the on-shell approximation. The use of the KMK kernel requires the introduction of a wavevector cut-off in the gap equation, a natural choice for which is discussed under “Methods” section.

Numerical calculations based on the full Eliashberg theory and the KMK kernel are found to be in close agreement as expected in the weak coupling limit^52^ and quite different from the predictions based on the on-shell approximation^53^. We stress that in weak coupling, the Eliashberg theory does not reduce to the BCS gap equation with the Fröhlich or Bardeen–Pines interaction used in standard textbooks. A correction to the KMK kernel has been shown to be important where the logarithm of *T*_c_ has a vanishing first-order term in the interaction strength^54,55^, a special case however that we find not to be relevant to SrTiO_3_.

It has been argued that a potentially serious challenge to the Eliashberg description can arise form vertex corrections in cases involving interactions mediated by bosons with energies well above the Fermi energy^54,56–58^. In light of these still poorly resolved effects we limit ourselves only to consider to what extent the model interaction, Eqs. (1)–(3), can account for the observed trend of *T*_c_ vs. density and pressure. For this purpose we restrict ourselves to the Eliashberg approximation applied in the weak coupling limit that is more accurate than the on-shell approximation. Also, we note that at carrier densities near to and above the superconducting dome maximum, the Fermi energy is comparable to the energies of the longitudinal hybrid polar modes that mediate the pairing interaction in the model defined by Eqs. (1)–(3).

The gap equation, Eq. (4), can be represented in operator form as

6 $${\Lambda} \;{\mathrm{{\Delta} }} = K{\mathrm{{\Delta} }}$$

where Δ is a vector and *K* is an operator whose matrix form is defined by Eq. (4) with Λ → 1. This is an eigenvalue equation and the eigenvalues can be found in the standard way by evaluating the determinant of the matrix *K* − Λ*I*, where *I* is the unit matrix. The transition temperature *T*_c_ is found by the condition that the highest eigenvalue, Λ_h_, is equal to unity.

Instead of determining *T*_c_ directly we could consider the behaviour of Λ_h_ in the low temperature limit as a function, in particular, of the carrier density and applied pressure. The region in density and pressure where superconductivity is expected to arise would be indicated by the condition Λ_h_ > 1. More generally, the maximum of Λ_h_ vs. density and pressure may be expected to indicate the regime where the contribution of the pairing interaction defined by Eqs. (1)–(3) to the total pairing interaction is strongest. Λ_h_ is sometimes considered a kind of susceptibility of the system to forming a superconducting state.

The details of the evaluation of the highest eigenvalue based on Eqs. (1)–(6) together with parameters representative for SrTiO_3_ are given under “Methods” section. Instead of presenting Λ_h_ vs. carrier density *n* and pressure *p* for a fixed low value of *T*, here we present results of the predicted *n* and *p* dependences of *T*_c_ inferred from the condition Λ_h_(*T* = *T*_c_, *n*, *p*) is equal to unity. This procedure depends on the known material properties and the cut-off in the sum defining the gap equation as noted above. Importantly, the effect of this cut-off is comparatively weak for the trends vs. *n* and *p* of normalized values of *T*_c_, and especially of normalized values of the highest eigenvalue Λ_h_. The trends of Λ_h_ in particular can hence be viewed as predictions of the model, Eqs. (1)–(6), that are essentially free of adjustable parameters.

We note that when calculating *T*_c_ it is often common practice to determine parameters “*λ*” (connected to the attraction between electrons) and “*μ*” (connected to the Coulomb repulsion of like charges). These effects are seamlessly incorporated into our model in the form of the interaction defined by Eqs. (1)–(3). The effects relating to the renormalization of “*μ*” to “μ*” in typical calculations are taken into account here by the form of the interaction and the calculation used to determine the gap function in Eqs. (4)–(6). This renormalization process is physically related to the fact that one of the particles in a two-particle pairing process is able to avoid the repulsive core of the other particle by changing its position as a function of space and time (sometimes referred to as retardation). In particular in the case of superconductivity in SrTiO_3_, the oscillations in time of the gap function and interaction function are synchronized such that electron repulsion may be mitigated. In terms of the frequency-dependent functions, this means that the gap function as a function of frequency changes sign when the interaction function becomes positive (repulsive). This can therefore be thought of as the frequency-space analogue of the phenomenon occurring in *k*-space (wavevector space) in magnetically mediated *d*-wave superconductors in which the gap function changes sign in *k*-space to help avoid particle repulsion.

**Fig. 4:**
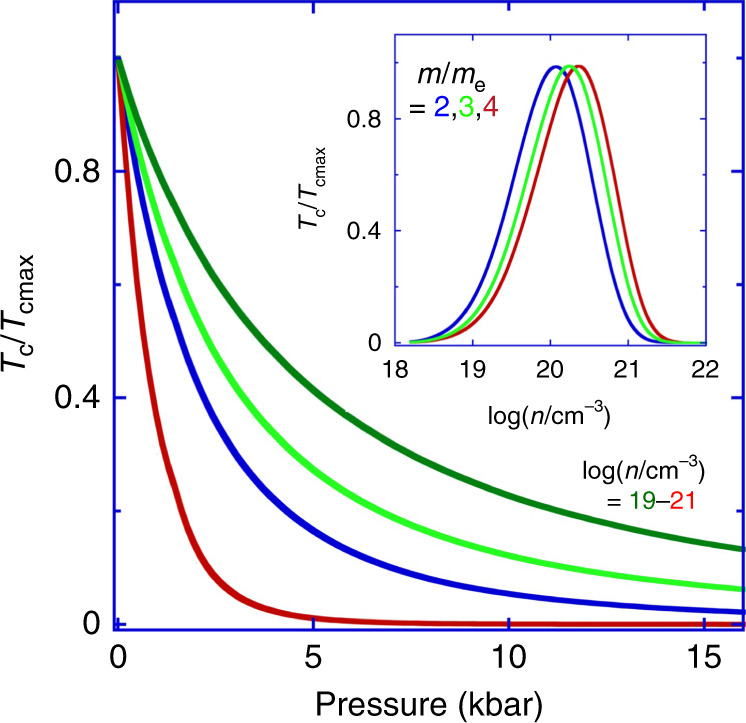
The calculated superconductivity phase diagram for Nb-doped SrTiO_3_. The normalized superconducting transition temperature, *T*_c_ calculated via the model given by Eqs. (1)–(5) and the material parameters relevant to SrTiO_3_ (see “Methods” section), is shown (i) vs. pressure, *p*, for values of *n* around the superconducting dome (Fig. 2a), log(*n*/cm^−3^) = 19, 20, 20.5, 21, from the upper to the lower curves, respectively, in the main figure, and (ii) vs. *n* at *p* = 0 for different values of the carrier mass, *m*/*m*_e_ = 2, 3 and 4, in the inset. *T*_cmax_ corresponds to the maximum of *T*_c_ at *p* = 0 in the main figure and the dome maximum in the inset. The ratio *T*_c_/*T*_cmax_ in contrast to *T*_c_ itself depends only weakly on the cut-off frequency used to evaluate Eqs. (1)–(5) (see “Methods” section and Fig. S3). The close correspondence with the observed phase diagram (Fig. 2a) suggests that the pairing mechanism modelled by Eqs. (1)–(3) plays a key role in superconductivity in SrTiO_3_. *T*_cmax_ is found to be in the mK range based on a realistic choice of parameters as outlined in the “Methods” section for SrTiO_3_ and as originating purely from the model described by Eqs. (1)–(3) in the main text and without a direct coupling to the transverse-optic ferroelectric critical modes.

The predicted variations of *T*_c_ vs. *n* and *p* are summarized in Fig. 4. From the inset we see that the calculated *T*_c_ initially rises with carrier density, *n*, reaches a maximum in the range 10^19^–10^20^ cm^−3^, and collapses at higher densities. This is in keeping with the trend of *T*_c_ seen experimentally^32^ as shown in the inset of Fig. 2a. We note that the KMK and Eliashberg analyses may be expected to overestimate the pairing strength in the over-doped regime so that in a better approximation the collapse of *T*_c_ at high *n* is expected to be more rapid than shown in the inset of Fig. 4, in closer agreement with experiments (see “Methods” section)^54,58^. Moreover, the absolute magnitude of *T*_c_, though only estimated to logarithmic accuracy by our calculations (see “Methods” section), suggests that the pairing mechanism we have considered here as defined by Eqs. (1)–(3), plays a dominant role in the formation of electron pairs at least near to optimal doping.

The relevance of this pairing mechanism (Eqs. (1)–(3)) is strikingly supported by the correspondence between the predicted and observed rapid collapse of *T*_c_ with increasing pressure and hence increasing 1/*ε*_0_ (Figs. 2 and 4). For densities in the range around 10^20^ cm^−3^ where the measurements were performed, *T*_c_ tends to increase with decreasing 1/*ε*_0_, i.e., as the ferroelectric QCP is approached (at fixed *n*, but variable lattice density; see “Methods” section). The effect of uniaxial stress on *T*_c_ may also be estimated via its effect on 1/*ε*_0_. 1/*ε*_0_ can increase or decrease depending on the direction of applied stress and whether it is compressive or tensile^6^. In cases where stresses lead to a decrease in 1/*ε*_0_ the model predicts an increase in *T*_c_. Experiments in which stress is varied result in anisotropies and inhomogeneities within samples, which could be the subject of future investigation. We note that the effects of isotropic or anisotropic biaxial strains in SrTiO_3_ films are of particular current interest and an important topic for future study (discussions may be found, for example, in refs. ^59–61^).

## Discussion

We have shown that the interaction described in terms of the dielectric function including the effects of fluctuations of the densities of both the ionic and conduction electrons can lead to a quantitative understanding of the doping and pressure dependence of the superconducting transition temperature of SrTiO_3_ and related materials. This is in manner similar to that previously proposed by Takada^41^, but including (i) a more tractable model for the interaction, (ii) a more transparent identification of the relevant longitudinal hybrid polar modes and limitations of the model Eqs. (1)–(4), (iii) a consideration of the cut-off wavevector in the gap equation, and (iv) comparisons with our new experiments as presented here. We do not rule out, however, other contributions to the total pairing interaction, which may be particularly important well away from optimal doping.

**Fig. 5:**
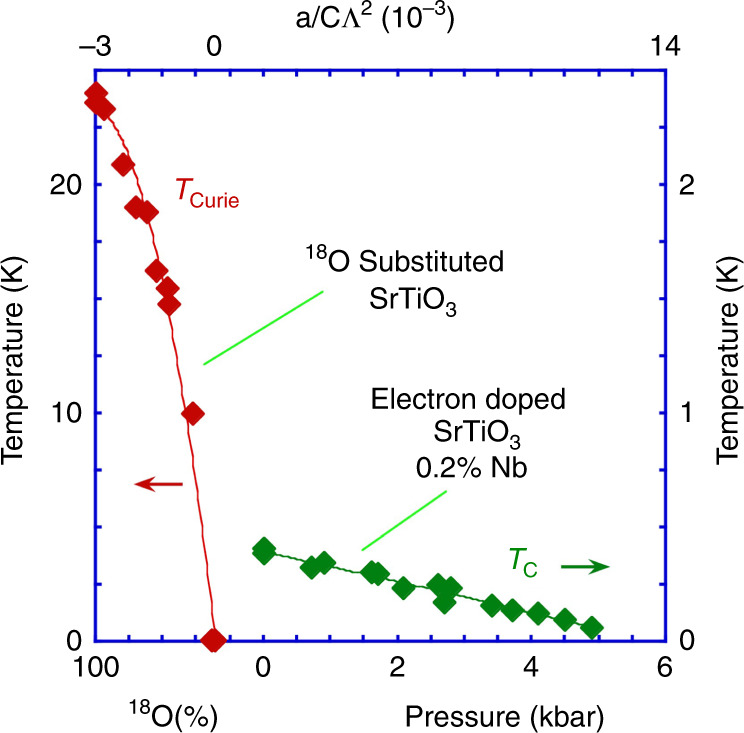
The overall observed phase diagram for SrTiO_3_ near to the ferroelectric quantum critical point. The top horizontal axis is the quantum tuning parameter referred to in Fig. 1 that may be calculated from the applied pressure or isotopic substitution (bottom horizontal axis). The parameters *a*, *c* and Λ defining the quantum tuning parameter *g* = *a*/(*c*Λ^2^) are, respectively, the coefficients of the quadratic and gradient-squared terms and the cut-off wavevector in the ϕ^4^ field theory^3^. *g* may be varied by pressure, chemical substitution, strain, isotope substitution, or charge-carrier density to tune between ferroelectric, paraelectric, and superconducting phases. From left to right, the Curie temperature collapses with increasing *g* and gives way to superconductivity in the presence of carrier doping. Note that at the carrier density given in the figure, the soft transverse-optical frequency and hence proximity to the QCP is not far from that in the undoped state^S34^ (see “Methods” section). The phase diagram is consistent with the model defined by Eqs. (1)–(5) for the superconducting transition, and with the model in ref. ^3^ for the Curie temperature, both calculated in terms of independently measured temperature-independent model parameters for SrTiO_3_.

For *n* not too far from optimal doping the model leads us to expect the occurrence of a superconducting dome in the temperature–lattice density phase diagram at fixed *n* with a maximum in the vicinity of the ferroelectric QCP (which in our case is predicted to occur at a small negative pressure). This is at least qualitatively similar to the behaviour observed in the phenomenon of superconductivity on the border, for example, of magnetic long-range order at low temperatures (see, e.g., ref. ^31^ and Fig. 5). The present model, however, predicts that the transition temperature may have a minimum rather than a maximum versus lattice density near to the ferroelectric QCP at doping levels much lower than optimal (see “Methods” section).

Some insight on this difference in behaviour can be obtained by considering the low and high *n* limits of the pairing interaction given by Eq. (3). From Eqs. (1)–(3) we find that in the low *q* and *ω* limit, and low *n* with *ω*_p_ << *ω*_+_ = Ω_LO_

7a $$V\left( {q,\omega } \right) = \frac{{4\pi e^2}}{{\varepsilon _\infty q^2}}\frac{{v_p^2}}{{\omega _{ {p}}^2}}\left( {1 - \frac{{v_{ {p}}^2}}{{v_{ {p}}^2 - \omega ^2}}} \right)$$

where *ν*_p_ = Ω(0)*ω*_p_/Ω_LO_ is the lower longitudinal hybrid polar mode frequency corresponding to the plasma oscillations associated with the charge carriers as screened by the ions. On the other hand, in the low *q* and *ω* limit and high *n* with Ω(*q*) << *ω*(*q*)

7b $$V\left( {q,\omega } \right) = \frac{{4\pi e^2}}{{\varepsilon _\infty (q^2 + k_{{ {TF}}}^2)}}\left( {1 - \frac{{v(q)^2}}{{v(q)^2 - \omega ^2}}} \right)$$

where *k*_*TF*_ is the Thomas–Fermi wavevector defined by *ω*(*k*_*TF*_) = *ω*_p_ and *ν*(*q*) = Ω_LO_*q*/*k*_*TF*_ is the lower longitudinal hybrid polar mode frequency corresponding in this case to vibrations of the ions as screened by the charge carriers. The prefactor in Eq. (7a) scales as Ω(0) and thus *V*(*q*,*ω*) tends to zero as Ω(0) tends to zero. Therefore, the pairing interaction at low *n* decreases with decreasing Ω(0) in the important low-energy regime, so that *T*_c_ may be expected to decrease with decreasing Ω(0*)*. There is no corresponding tendency in the high *n* regime, in which the pairing interaction in the limit considered reduces to the Bardeen and Pines interaction for jellium (Eq. (7b)). In the intermediate regime relevant to our measurements both of the longitudinal hybrid polar modes are important and we have appealed to numerical analyses to demonstrate that *T*_c_ tends to increase with decreasing Ω(0) in this regime, as observed.

Thus, we anticipate a dramatic qualitative change in form of the pressure dependence of Λ_h_ (the propensity of the system to forming a superconducting state) as a function of carrier density (see Supplementary Information). The change in behaviour is however expected to occur for carrier densities below 10^18^ cm^−3^, where our Nb-doped SrTiO_3_ samples are not superconducting down to at least 0.04 K, the lowest temperatures investigated. Superconductivity has been reported in oxygen-depleted specimens for carrier densities somewhat below 10^18^ cm^−3^, but the role of inhomogeneities in these systems complicates the interpretation of the experimental results.

Along with this predicted qualitative change in behaviour with carrier density, our analyses differ, e.g., from that of refs. ^20,44^ by focusing on pairing via the virtual exchange of two longitudinal hybrid polar modes rather than the critical TO mode, and by explicitly including: (i) the Coulomb repulsion between carriers, (ii) screening due to added carriers, (iii) dynamics of the KMK versus on-shell kernel, and (iv) the retardation effect explicitly. This allows us to calculate the superconducting dome structure without the use of adjustable parameters in terms of a direct repulsion and an indirect dielectric interaction mediated via the exchange of two longitudinal hybrid polar modes in place of the critical mode alone as in, e.g., ref. ^20^.

We would like to highlight the following features of the superconductivity as observed and modelled in Nb-doped SrTiO_3_. Firstly, around optimal doping, the Fermi energy is not particularly small compared to the hybrid polar mode frequencies (see Fig. 3a). The attraction between electrons is facilitated over a range of frequencies and in particular for frequencies close to two hybrid-polar-mode frequencies, one a little below the Fermi Energy and one a little above the Fermi energy. To understand the effects of such an interaction potential on superconductivity, it is beneficial to appeal to detailed quantitative calculations for insight as presented here. It is notable that the strength of the interaction, *V*, is substantial due to the fact that it originates from polar fluctuations in a highly polarizable medium that have to a great extent escaped conduction-electron screening. As explained earlier, the phenomenon of retardation is incorporated into our description of superconductivity in two important ways. Firstly, in the retardation of the frequency-dependent interaction *V*, and secondly in the retardation of the frequency-dependent gap function *Δ* (the superconducting order parameter). Both of these retardation effects greatly assist the formation of the superconducting state along with the strong polar fluctuation-mediated interaction.

The analysis presented here highlights the crucial importance of ionic polarizability in yielding an extraordinarily strong pairing interaction leading to pair formation via ‘ion mediation’, and may help our understanding of other superconductors with electron–polar phonon coupling^62^. Indeed, this interaction may be thought of as leading to a third generation of superconductors. The first generation being traditional phonon-mediated superconductivity (involving vibrations of approximately neutral atoms), the second being magnetically mediated superconductivity (involving the exchange energy, *J*) and the third involving strong attraction mediated by polar fluctuations. We have identified that the predominant contribution to the formation of Cooper pairs originates from induced interactions arising from the effects of the coupled conduction-electron and polar-phonon system and described here as longitudinal hybrid-polar-modes. Such a model may also find applications in superconductors where the conduction electrons are coupled to carriers originating from a separate hole pocket rather than to a separate ionic system, such as that recently considered in low carrier-concentration semi-metals^63^. There may exist circumstances under which the advantageous increase in the Fermi energy with increasing carrier density is not offset by a loss in interaction strength between the carriers, in the way implied by Eqs. (1)–(3), potentially leading to pair formation at elevated temperatures as in the related perovskite oxide BaBiO_3_ doped with potassium (see, e.g., ref. ^62^ and references therein).

*Comments*: Since this work was completed, further experimental studies^34,64–66^ and theoretical studies^67–69^ have been reported that are generally in keeping with our conclusions. A theoretical expression for the integration cut-off in the Eliashberg gap equation has been discussed^69^ and leads to numerical values of *ω*_c_ for SrTiO_3_ of the same order of magnitude as those used in our analyses in the density range near to the dome maximum (see also “Methods” section and Supplementary Information). A review of superconductivity in carrier-doped SrTiO_3_ has been given in ref. ^47^.

## Methods

### Experimental

Incipient ferroelectric SrTiO_3_ may be doped into a metallic state by a number of methods including oxygen reduction and niobium substitution. Metallic specimens of SrNb_*x*_Ti_1−*x*_O_3_ were obtained from commercial sources with niobium nominal doping of 0.02, 0.2, and 1 at.%, corresponding to nominal charge-carrier concentrations, *n*, with log(*n*/cm^−3^) of 18.5, 19.5, and 20.3, respectively. The samples were cut into parallelepipeds with approximate dimensions 4 mm × 1 mm × 0.5 mm. Low-resistance Ohmic contacts were achieved by etching the surfaces using argon-ion plasma followed by sputtering of gold contacts over a titanium seed-layer on the top surfaces in a standard Hall bar geometry. Measurements of the Hall resistance *R*_*xy*_ at liquid-helium temperatures in a field up to 9 T yield log(*n*/cm^−3^) of the order of 18.0, 19.3, and 20.6 in fair agreement with the nominal values for the three samples, respectively. The value of *T*_c_ for the sample with 0.2 at.% Nb is close to the maximum expected at log(*n*/cm^−3^) of order 20 (inset of Fig. 2a), the difference from the nominal value of 19.5 perhaps being due to doping inhomogeneities. Four-terminal resistance *R*_*xx*_ was measured in zero-field for each sample at ambient pressure down to 50 mK using an adiabatic demagnetization refrigerator. The residual resistance ratio, defined as the resistance at room temperature divided by the resistance at 2 K, was >600 for all samples.

The 0.2 at.% Nb sample with the highest superconducting transition temperature *T*_c_ = 0.4 K was selected for the high-pressure experiments. To check for repeatability, low-temperature resistivity measurements under hydrostatic pressures were carried out on two different adiabatic demagnetization refrigerators, one in Cambridge and one in Rio de Janeiro, using two different piston-cylinder clamp cells. In these experiments, hydrostatic pressure was applied to the sample at room temperature using fluorinert (1:1, FC84–FC87) as the pressure-transmitting fluid. For each fixed pressure, four-terminal resistance of the sample was measured using a Cambridge Cryogenics mK measurement system with a lock-in amplifier and constant current source as a function of temperature down to 50 mK. The pressure was determined in the low-temperature range by measuring the superconducting transition temperature of a high-purity tin manometer. For all observations of superconductivity in our SrNb_*x*_Ti_1−*x*_O_3_ samples, we defined *T*_c_ to be the temperature at which the resistivity dropped by 10% from its value on entering the superconducting state. Measurements were collected during cooling and heating runs at a rate of ~1 K/h. Estimates of the uncertainties in our determinations of pressure, *T*_c_ and the *T*^2^ coefficient of the normal state resistivity, *A*, are indicated by the error bars (or data point sizes) in Fig. 2.

### Theoretical

*The longitudinal hybrid frequencies and coupling parameters*: The hybrid longitudinal mode frequencies, *ω*_±_(*q*), and coupling functions, *γ*_±_(*q*), can be determined in terms of the starting model parameters in *ε*(*q*, *ω*) by writing the sum of unity and two resonances on the right-hand side of Eq. (2) as a ratio of two fourth-order polynomials, each of which can be factorized in terms of two second-order polynomials. Applying the same procedure to 1/*ε*(*q*, *ω*) expressed as a sum of unity and two resonances (Eqs. (1) and (3)) leads to the following closed-form expressions for *ω*_±_(*q*) and *γ*_±_(*q*) in terms of the starting model parameters in Eqs. (2a)–(2c)

8a $$\omega _ \pm (q) 	= \frac{1}{2}\left( {\omega \left( q \right)^2 \,\, + \,\, {\mathrm{{\Omega} }}\left( q \right)^2 \,\, + \,\, \omega _{\rm{{p}}}^2 \, + \, {\mathrm{{\Omega} }}_{\rm{{p}}}^2} \right)\\ 	\quad\pm \left( {\frac{1}{4}\left( {\omega (q)^2 + {\mathrm{{\Omega} }}(q)^2 + \omega _{\rm{{p}}}^2 + {\mathrm{{\Omega} }}_{\rm{{p}}}^2} \right)^2 \, - \, \omega \left( q \right)^2{\mathrm{{\Omega} }}_{\rm{{p}}}^2 - {\mathrm{{\Omega} }}\left( q \right)^2\omega _{\rm{{p}}}^2 - \omega (q)^2{\mathrm{{\Omega} }}(q)^2} \right)^{1/2}$$

8b $$\gamma _ \pm (q) = \left( {\omega \left( q \right)^2 \,\, + \,\, {\mathrm{{\Omega} }}\left( q \right)^2 \,\, - \,\, \omega _ \pm \left( q \right)^2 \, - \, \omega (q)^2{\Omega} (q)^2\omega _ \pm \left( q \right)^{ - 2}} \right)/\left( {\omega _ + \left( q \right)^2 \, - \, \omega _ - \left( q \right)^2} \right)$$

*The KMK kernel*: Each resonance factor in the interaction function *V*(*q*, *ω*) (Eq. (3))

9a $$\frac{{\omega _ \pm \left( q \right)^2}}{{\omega _ \pm \left( q \right)^2 \, - \, \omega ^2}}$$

is replaced in the interaction kernel *U*(*k*, *k*′) in the BCS gap equation (Eq. (4)) by a factor of the form

9b $$\frac{{\omega _ \pm \left( {k{^\prime} - k} \right)^2}}{{\omega _ \pm \left( {k{^\prime} - k} \right)^2 - \left( {\xi \left( {k{^\prime}} \right) - \xi \left( k \right)} \right)^2}}$$

in the on-shell approximation, or

9c $$\frac{{\omega _ \pm \left( {k{^\prime} - k} \right)}}{{\omega _ \pm \left( {k{^\prime} - k} \right) + \left| {\xi \left( {k{^\prime}} \right)} \right| + \left| {\xi \left( k \right)} \right|}}$$

in the KMK approximation^40^. Note that in the KMK approximation the kernel falls off with increasing values of the single-particle energies individually and not their difference as in the incompletely physical case of the on-shell approximation. The KMK kernel is derived from the Eliashberg equation referred to in the main text (Eq. (5)) in the weak-coupling limit appropriate to SrTiO_3_.

*Parameters used in numerical calculations*: The calculation presented in Figs. 3 and 4 and in Supplementary Information are based on Eqs. (1)–(6), (8) and (9c) (KMK kernel) in the effective mass approximation. Except where otherwise indicated the model parameters employed are estimated from independent experiments in the low-temperature limit: *ε*_∞_ = 5, *ε*_0_ = 2.5 × 10^4 3,S42^, *m* = 4*m*_e_^S25,S35^ (relevant near to optimal doping), Ω_LO_ = 100 meV ^S34^ (see caption of Fig. 3), *v*_s_ = 5 meVÅ ^S33^ and

10 $${\mathrm{{\Omega} }}\left( 0 \right) = {\mathrm{{\Omega} }}_{{\rm{{LO}}}}\left( {\frac{{\varepsilon _\infty }}{{\varepsilon _0}}\left( {1 + \frac{n}{{{\mathrm{{\Delta} }}n}}} \right)\left( {1 + \frac{p}{{{\mathrm{{\Delta} }}p}}} \right)} \right)^{1/2}$$

where Δ*n* = 1.0 × 10^19^ cm^−3^ and Δ*p* = 0.7 kbar (see main text for further relevant references to the literature). The solution of the gap equation also involves an upper cut-off wavevector or its corresponding frequency taken to be 4Ω_LO_. The cut-off affects the magnitude of *T*_c_ or more generally the highest eigenvalue Λ_h_ in Eq. (6), but does not significantly affect the qualitative dependence of Λ_h_ on the carrier density, *n*, or pressure, *p* (see also Supplementary Information). The cut-off discussed recently^69^ by taking into account the effects of quasiparticle damping is in the range of cut-offs considered in Supplementary Information. The increments in the sums defining the gap equation were reduced in size until the results reached essential convergence. Calculations employing the full Lindhard function, and hence dissipation in the single particle continuum, give qualitatively similar results for the upper eigenvalues where comparisons have been made. In contrast to *T*_c_ itself, Λ_h_, and the position and width in *n* and *p* of the predicted superconducting dome, are seen to be comparatively weakly dependent on the choices of parameters. Hence the results form quite robust features of the model presented here (Figs. 3 and 4 and Supplementary Information).

## Supplementary information

Supplementary Information

## Data Availability

The data supporting the findings of this study are available within the paper. Any additional data connected to the study are available from the corresponding author upon reasonable request.
