# Author Correction: Superconductivity mediated by polar modes in ferroelectric metals

**DOI:** 10.1038/s41467-020-19239-1

**Published:** 2020-10-15

**Authors:** C. Enderlein, J. Ferreira de Oliveira, D. A. Tompsett, E. Baggio Saitovitch, S. S. Saxena, G. G. Lonzarich, S. E. Rowley

**Affiliations:** 1 Cavendish Laboratory, University of Cambridge, J. J. Thomson Avenue, Cambridge, CB3 0HE UK; 2 Centro Brasileiro de Pesquisas Físicas, Rua Dr Xavier Sigaud 150, Rio de Janeiro, 22290-180 Brazil; 3 UFRJ, Estrada de Xerém 27, Xerém, Duque de Caxias, Rio de Janeiro, 25245-390 Brazil; 4 National University of Science and Technology MISiS, Leninsky Prospekt 4, Moscow, 119049 Russia

**Keywords:** Electronic devices, Ferroelectrics and multiferroics, Phase transitions and critical phenomena, Superconducting properties and materials

Correction to: *Nature Communications* 10.1038/s41467-020-18438-0, published online 25 September 2020.

The original version of this Article contained an error in the caption of Fig. 1, which incorrectly says “SrTi(^16^O_1−*y*_^18^O_*y*_)_3_”, but in fact it should read “SrTi(^16^O_1−*x*_^18^O_*x*_)_3_”.

