# Supplementary material for: Superconductivity mediated by polar modes in ferroelectric metals
Source: Nat Commun. 2020 Sep 25;11:4852. doi: 10.1038/s41467-020-18438-0 (PMC7519043; doi:10.1038/s41467-020-18438-0)
Supplement: Supplementary file 1 — Supplementary Information [file 41467_2020_18438_MOESM1_ESM.pdf]

Supplementary Information

Superconductivity mediated by polar modes in ferroelectric metals

C. Enderlein[1,2,3], J. Ferreira de Oliveira[1,2], D. A. Tompsett[1], E. Baggio Saitovitch[2], S. S. Saxena[1,4], G. G. Lonzarich[1] and S. E. Rowley[1,2]

1. Cavendish Laboratory, University of Cambridge, J. J. Thomson Avenue, Cambridge, CB3 0HE, United Kingdom
2. Centro Brasileiro de Pesquisas Físicas, Rua Dr Xavier Sigaud 150, Rio de Janeiro, 22290-180, Brazil
3. UFRJ, Estrada de Xerém 27, Xerém, Duque de Caxias, Rio de Janeiro, 25245-390, Brazil
4. National University of Science and Technology MISiS, Leninsky Prospekt 4, Moscow 119049, Russia

Correspondence to: Dr. Stephen E. Rowley, ser41@cam.ac.uk

In Supplementary Figure S1 we present results showing resistive superconducting transitions in Nb-doped samples of strontium titanate ($SrTi_{1-x}Nb_xO_3$) (inset) with carrier densities, n, below, near to and above the maximum of the superconducting dome in the T_c vs. n phase diagram (Fig. 2 of the main paper), and the form of the temperature dependence of the resistivity (main figure) for the sample with the highest value of T_c.

In Supplementary Figure S2 we present models of the Fermi surface of $SrTiO_3$ for carrier densities across the superconducting dome as defined above.

In Supplementary Figures S3-S6 we present calculations of the maximum eigenvalue as defined by the KMK-Eliashberg gap equation, which measures the strength of Cooper pair formation in the model for $SrTiO_3$ described in the main text. The figures compare the effects of varying the chief model parameters: the cut-off frequency ω_c, the TO frequency (as defined by Eq. 10 (main text) in terms of the carrier density, pressure, ε_0 of the starting undoped system (i.e of $SrTiO_3$)), the starting LO frequency and the carrier mass.

In Supplementary Figure S7 we compare the form of the normalized pairing interaction as a function of real and imaginary frequencies for the simplest case of a single resonance mode to clarify how the two representations reflect the pairing tendency. A more realistic case is presented in Fig. 3 of the main paper.

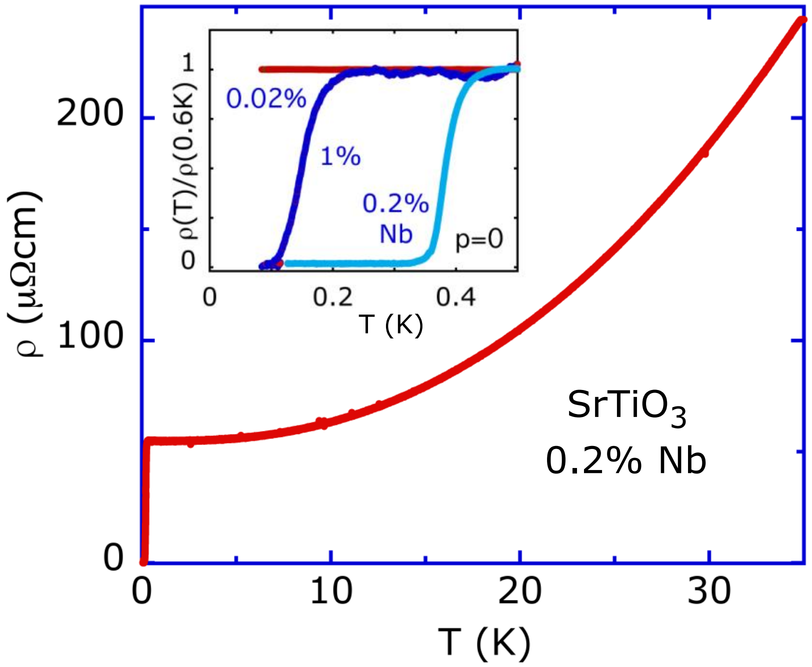

Figure S1. Temperature dependence of the resistivity of Nb-doped $SrTiO_3$ around optimal doping (see Ref. 3 main text). A fit to a function of the form $\rho = \rho_0 + AT^x$ yields an exponent x close to 2. In agreement with previous observations[32,71,72] the exponent is well below that normally expected in terms of the conventional theory of electron-phonon scattering. Note that an exponent of 2 originating from the conventional Fermi-liquid theory of metals is not expected to apply in the case of doped $SrTiO_3$ over the range of temperatures measured due to its low charge-carrier density. The inset shows the resistivity normalized to its 0.6K value against temperature for three doping levels investigated, with nominally 0.02, 0.2 and 1 at% Nb, *i.e.*, below, near to and above optimal doping, respectively, *i.e.*, for n around the maximum of T_c vs. n, which is observed at approximately 10^{20} cm^{-3} (Fig. 1a and Methods in main text). Note that superconductivity has not been reported for Nb substituted samples for carrier densities lower than of the order of 10^{18} cm^{-3}.

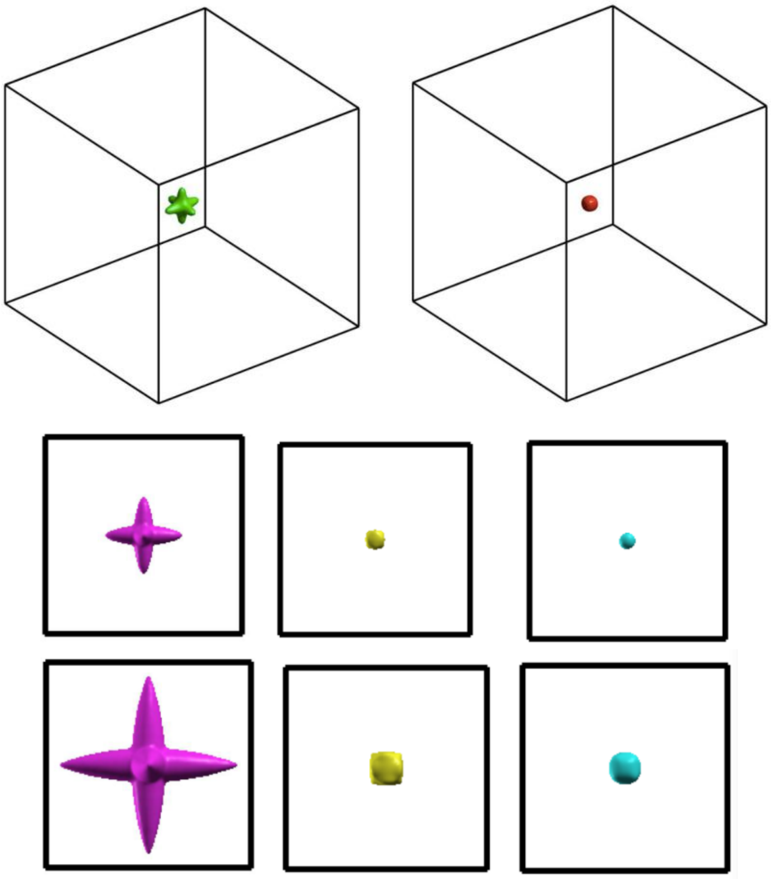

Figure S2. The Fermi surface calculated using Density Functional Theory (DFT) for carrier-doped $SrTiO_3$, with *n* of approximately 10^{19} cm^{-3} (upper figure) showing the full Brillouin zone, 10^{20} cm^{-3} (middle panel), and 10^{21} cm^{-3} (lower panel). Two to three of the three t2g bands are populated yielding the electron pockets of the Fermi surface shown. The lower band with the fluted Fermi surface dominates the density of states at the Fermi level (see Fig. 3 in the main text for the Fermi energy vs. carrier density). The boundaries of the first Brillouin zone of $SrTiO_3$ in its undistorted cubic lattice structure are indicated. The density functional technique employed in the calculations was carried out using the all-electron Wien2k code, with an augmented plane wave (APW) basis using the local density approximation (LDA). Muffin tin radii were set to 2.36, 1.72 and 1.54 a.u. for Sr, Ti and O, respectively, with RKMAX = 7. The ambient pressure low *T* experimental lattice parameter was used with 3375 k-points in the full Brillouin zone. For this illustration, carrier doping was achieved via a rigid shift of the Fermi level.

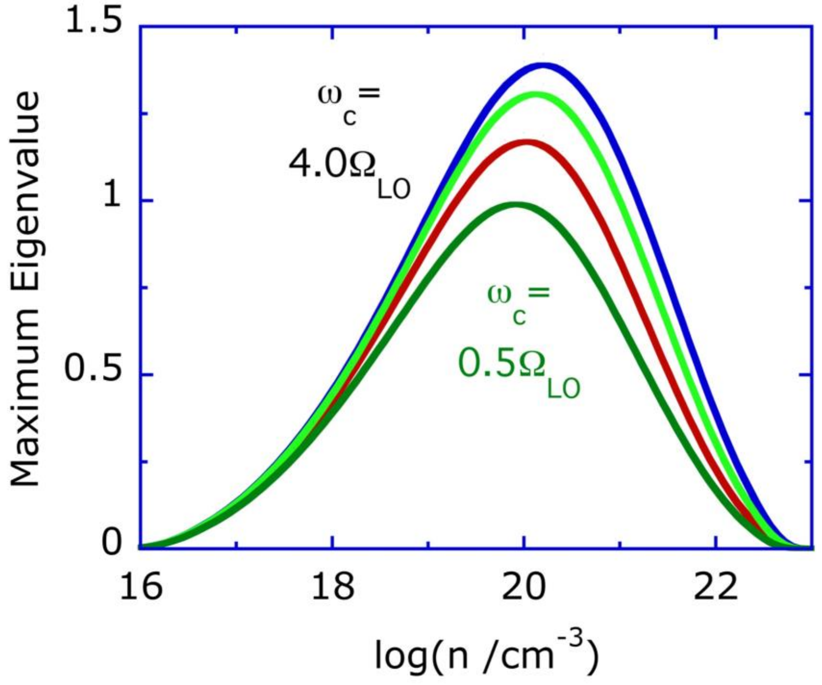

Figure S3. The maximum eigenvalue, Λ_h, of the KMK-Eliashberg gap equation calculated via the model described in the main text and under Methods for different values of the cut-off frequency $\omega_c = 0.5, 1, 2$ and $4\ \Omega_{LO}$. The parameters entering the calculations are given under Methods and the calculations are carried out for a reference temperature of 10^{-2} K. The peak positions and widths of the domes of the maximum eigenvalue vs. carrier density are relatively insensitive to the choice of the cut-off frequency over a sizeable range.

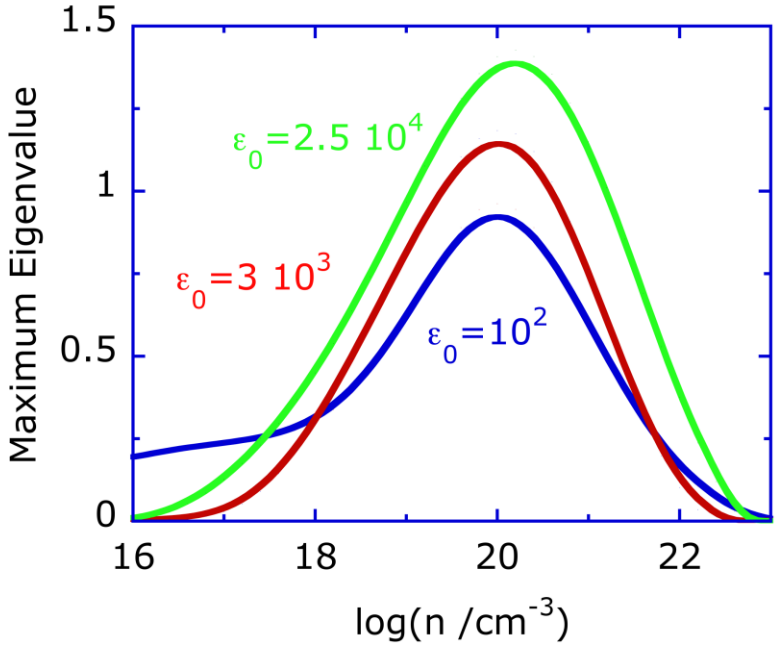

Figure S4. The maximum eigenvalue, Λ_h, of the KMK-Eliashberg gap equation, calculated for a reference temperature of 10^{-2} K, via the model described in the main text and under Methods, except for the different values of ε_0 as labelled in the figure. The maximum eigenvalue is seen to increase with increasing ε_0 in the range 10^{18} cm^{-3}-10^{22} cm^{-3}, in agreement with observation (Fig. 2a, main text). In the range 10^{16} cm^{-3}-10^{17} cm^{-3}, however, we observe inverted behaviour (at least for the widely separated values of ε_0 given in the figure), qualitatively as predicted recently by other techniques (see refs. 20 and 22, main text).

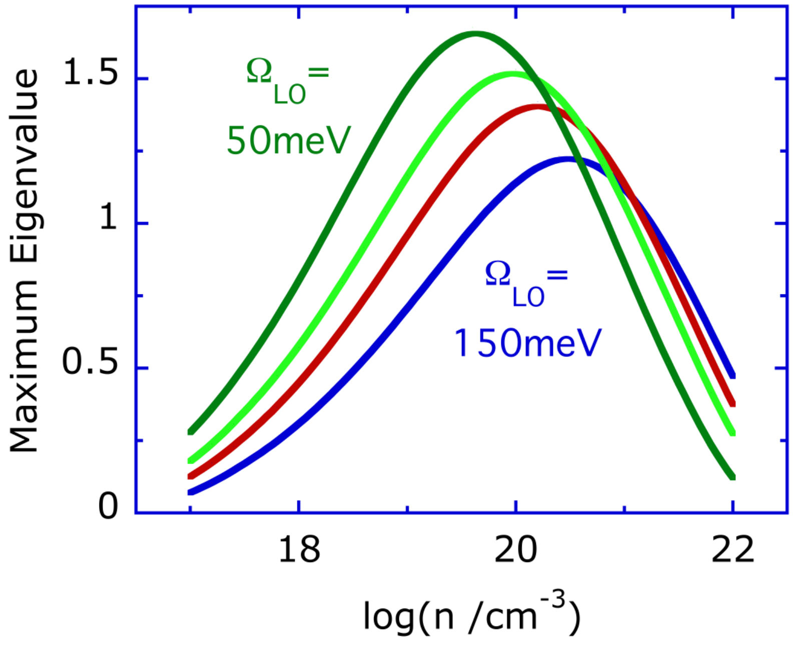

Figure S5. The maximum eigenvalue, Λ_h, of the KMK-Eliashberg gap equation calculated via the model described in the main text and under Methods for different values of the starting LO phonon frequency Ω_{LO} = 50, 75, 100 and 150 meV from the upper to the lower curves. The parameters entering the calculations are as given under Methods except for the different values of Ω_{LO} and the calculations are carried out for a reference temperature of 10^{-2} K. The peak position increases with increasing LO frequency. As shown in the inset of Fig. 4 (main text) the peak position also increases with increasing carrier mass m/m_e. These are examples of predictions that can be used to test (in principle falsify) the present description of superconductivity in $SrTiO_3$ and perhaps help to "design" new superconductors.

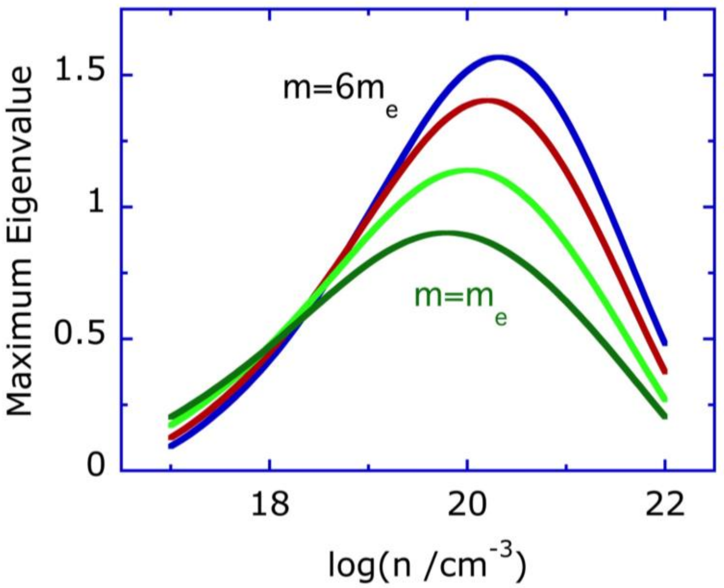

Figure S6. The maximum eigenvalue, Λ_h, of the KMK-Eliashberg gap equation calculated via the model described in the main text and under Methods for different values of the starting carrier mass m = 1, 2, 4, and 6 m_e from the lower to the upper curves. The parameters entering the calculations are as given under Methods except for the different values of m and the calculations are carried out for a reference temperature of 10^{-2} K. The peak increases in position and size with increasing m. As shown in Fig. S5 the peak position increases while the size decreases with increasing LO frequency Ω_{LO}. These are examples of predictions that can be used to test (in principle falsify) the present description of superconductivity in $SrTiO_3$ and perhaps help to "design" new superconductors.

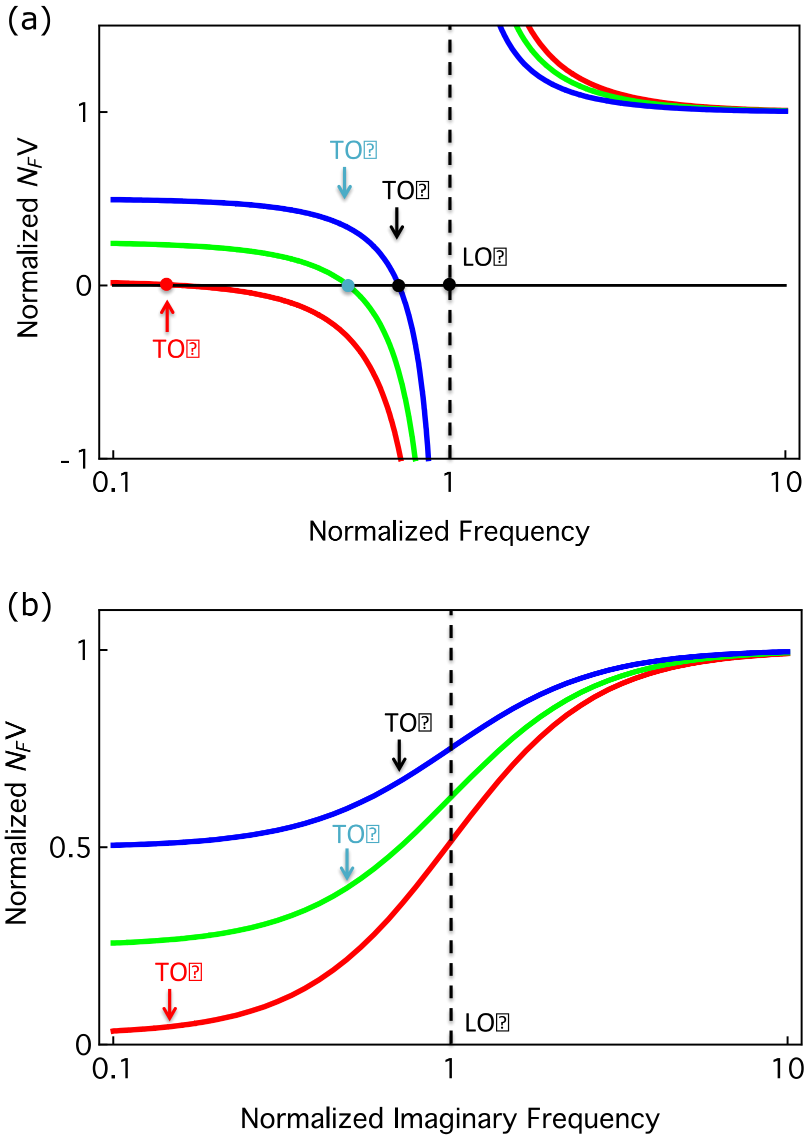

Figure S7. The characteristic forms of the normalized interaction function $N_F V(q,\omega)$ vs. frequency normalized to the LO frequency in the limit n & $q \rightarrow 0$ for different values of ε_0 and hence of the TO frequency. (a) As a function of real frequency the range of the attractive interaction in the interval between the TO and LO frequencies increases with increasing ε_0 (hence decreasing TO frequency) and extends down to zero frequency at the ferroelectric quantum critical point. (b) As a function of imaginary frequency as employed in the KMK-Eliashberg theory the magnitude of the attraction is reflected in the size of the step-like increase of the interaction function between the TO and LO frequencies which grows as the ferroelectric quantum critical point is approached. The corresponding description in the presence of charge carriers is more complicated but conceptually related (see caption of Fig. 4 in the main text). Although the interaction function vanishes at the TO frequency, a decrease in the TO frequency increases the frequency range of attraction, while attenuating the frequency range of repulsion at low frequencies. This leads to an enhancement of pair formation as a ferroelectric quantum critical point is approached, even without direct coupling to the soft critical TO mode. A similar description (but different in

detail) might apply in the case of magnetically mediated pairing where the softening of the critical mode at a magnetic quantum critical point appears, in a number of examples, to enhance pairing, even though the direct coupling to the soft critical mode itself may be expected to be impaired by quasiparticle damping (see, e.g., ref. 30 in the main text and references cited therein).

Supplementary References

S1 Anderson, P. W. Qualitative Considerations on the Statistics of the Phase Transition in $BaTiO_3$-type Ferroelectrics. *Conf. Proc. Lebedev Physics Inst. Acc. Sci. USSR. Fizika Dielektrikov*, 290-297 (1958).

S2 Cochran, W. Crystal stability and the theory of ferroelectricity. *Physical Review Letters* **3**, 412 (1959).

S3 Cochran, W. Crystal stability and the theory of ferroelectricity. *Adv Phys* **9**, 387-423 (1960).

S4 Vaks, V., GALITSKIT, V. & Larkin, A. Collective Excitations Near Second-order Phase-transition Points. *SOVIET PHYSICS JETP* **24** (1967).

S5 Vaks, V., Larkin, A. & Pikin, S. Self-consistent field method in the description of phase transitions. *Zh. Eksp. Teor. Fiz* **51** (1967).

S6 Rechester, A. B. Contribution to the theory of second-order phase transitions at low temperatures. *Sov. Phys. JETP* **33**, 423 (1971).

S7 Khmelnitskii, D. E. & Shneerson, V. L. Low-temperature displacement-type phase transition in crystals. *Sov. Phys. - Solid State* **13**, 687 (1971).

S8 Khmelnitskii, D. E. & Shneerson, V. L. Phase transition of the displacement type in crystals at very low temperature. *Sov. Phys. JETP* **37**, 164 (1973).

S9 Schneider, T., Beck, H. & Stoll, E. Quantum Effects in an N-Component Vector Model for Structural Phase-Transitions. *Physical Review B* **13**, 1123-1130 (1976).

S10 Bilz, H., Benedek, G. & Bussmannholder, A. Theory of Ferroelectricity - the Polarizability Model. *Physical Review B* **35**, 4840-4849 (1987).

S11 Roussev, R. & Millis, A. J. Theory of the quantum paraelectric-ferroelectric transition. *Physical Review B* **67**, 014105 (2003).

S12 Das, N. & Mishra, S. G. Fluctuations and criticality in quantum paraelectrics. *Journal of Physics: Condensed Matter* **21**, 095901 (2009).

S13 Palova, L., Chandra, P. & Coleman, P. Quantum critical paraelectrics and the Casimir effect in time. *Physical Review B* **79**, 075101 (2009).

S14 Rowley, S. E. *et al.* Quantum criticality in a uniaxial organic ferroelectric. *Journal of Physics: Condensed Matter* **27**, 395901 (2015).

S15 Rowley, S. E. *et al.* Uniaxial ferroelectric quantum criticality in multiferroic hexaferrites $BaFe_{12}O_{19}$ and $SrFe_{12}O_{19}$. *Scientific Reports* **6**, (2016).

S16 Rowley, S. E. *Quantum Phase Transitions in Ferroelectrics* PhD thesis, University of Cambridge, (2010).

S17 Coak, M. J. *Quantum Tuning and Emergent Phases in Charge and Spin Ordered Materials* PhD thesis, University of Cambridge, (2017).

S18 Rowley, S. E. & Lonzarich, G. G. Ferroelectrics in a twist. *Nature Physics* **10**, 907-908 (2014).

S19 Höchli, U. & Boatner, L. Quantum ferroelectricity in $K_{1-x}Na_xTaO_3$ and $KTa_{1-y}Nb_yO_3$. *Physical Review B* **20**, 266 (1979).

S20 Peercy, P. S. & Samara, G. A. Pressure and Temperature Dependences of Dielectric Properties and Raman-Spectra of RbH_2PO_4. *Physical Review B* **8**, 2033-2048 (1973).

S21 Horiuchi, S. *et al.* Quantum ferroelectricity in charge-transfer complex crystals. *Nat Commun* **6**, (2015).

S22 Kvyatkovskii, O. E. Quantum Effects in Incipient and Low-Temperature Ferroelectrics (A Review). *Phys Solid State+* **43**, 1401–1419 (2001).

S23 Lin, X., Zhu, Z., Fauqué, B. & Behnia, K. Fermi surface of the most dilute superconductor. *Physical Review X* **3**, 021002 (2013).

S24 Lin, X. *et al.* Critical doping for the onset of a two-band superconducting ground state in $SrTiO_{3-\delta}$. *Physical Review Letters* **112**, 207002 (2014).

S25 van der Marel, D., van Mechelen, J. L. M. & Mazin, I. I. Common Fermi-liquid origin of T-2 resistivity and superconductivity in n-type $SrTiO_3$. *Physical Review B* **84** (2011).

S26 Cohen, M. L. Superconductivity in many-valley semiconductors and in semimetals. *Physical Review* **134**, A511 (1964).

S27 Schooley, J. F. *et al.* Dependence of the superconducting transition temperature on carrier concentration in semiconducting $SrTiO_3$. *Physical Review Letters* **14**, 305-307 (1965).

S28 Eagles, D. Polaron coupling coustants in $SrTiO_3$. *J Phys Chem Solids* **26**, 672 (1965).

S29 Koonce, C., Cohen, M. L., Schooley, J., Hosler, W. & Pfeiffer, E. Superconducting Transition Temperatures of Semiconducting $SrTiO_3$. *Physical Review* **163**, 380 (1967).

S30 Eagles, D. Predicted Transition Temperatures of Very Thin Films and Whiskers of Superconducting Semiconductors—Application to $SrTiO_3$. *Physical Review* **164**, 489 (1967).

S31 Eagles, D. Predicted Transition Temperatures of Very Thin Films and Whiskers of Superconducting Semiconductors—Application to $SrTiO_3$. *Physical Review* **183**, 608 (1969).

S32 Scott, J. F. Soft-Mode Spectroscopy - Experimental Studies of Structural Phase-Transitions. *Rev Mod Phys* **46**, 83-128 (1974).

S33 Yamada, Y. S., G. Neutron Scattering and Nature of the Soft Optical Phonon in $SrTiO_3$. *J Phys Soc Jpn* **26**, 396-403 (1969).

S34 Van Mechelen, J. L. M. *Charge and Spin electrodynamics of $SrTiO_3$ and $EuTiO_3$ studied by optical spectroscopy* PhD thesis, Université de Genève, (2010).

S35 Van Mechelen, J. *et al.* Electron-phonon interaction and charge carrier mass enhancement in $SrTiO_3$. *Physical Review Letters* **100**, 226403 (2008).

S36 Pines, D. Electron interaction in solids. *Canadian Journal of Physics* **34**, 1379-1394 (1956).

S37 Ng, T. & Varma, C. Ferroelectrics with Low-energy Electronic Excitations. *arXiv preprint cond-mat/0102050* (2001).

S38 Bardeen, J., Cooper, L. N. & Schrieffer, J. R. Theory of superconductivity. *Physical Review* **108**, 1175 (1957).

S39 Bogoliubov, N. A NEW METHOD IN THE THEORY OF SUPERCONDUCTIVITY. 1. *Sov Phys Jetp-USSR* **7**, 41-46 (1958).

S40 Morel, P. & Anderson, P. Calculation of the superconducting state parameters with retarded electron-phonon interaction. *Physical Review* **125**, 1263 (1962).

S41 Eliashberg, G. Interactions between electrons and lattice vibrations in a superconductor. *Sov. Phys. JETP* **11**, 696-702 (1960).

S42 Muller, K. A. & Burkard, H. $SrTiO_3$ - Intrinsic Quantum Para-Electric Below 4-K. *Phys. Rev. B* **19**, 3593-3602 (1979).
